# Supplementary material for: Dickkopf1 fuels inflammatory cytokine responses
Source: Commun Biol. 2022 Dec 20;5:1391. doi: 10.1038/s42003-022-04368-8 (PMC9765382; doi:10.1038/s42003-022-04368-8)
Supplement: Supplementary file 1 — Supplemental Material and Methods [file 42003_2022_4368_MOESM1_ESM.pdf]

Supplementary Materials for

Dickkopf1 fuels inflammatory cytokine responses

Nikolai P. Jaschke[1*], Sophie Pählig[1], Anupam Sinha[1,2], Timon E. Adolph[3], Maria Ledesma Colunga[1], Maura Hofmann[1], Andrew Wang[4,5], Sylvia Thiele[1], Julian Schwärzler[3], Alexander Kleymann[6], Marc Gentzel[7], Herbert Tilg[3], Ben Wielockx[2], Lorenz C. Hofbauer[1], Martina Rauner[1], Andy Göbel[1], Tilman D. Rachner[1]

[1]Department of Medicine III & Center for Healthy Aging, Technische Universität Dresden, Dresden, Germany
[2]Institute of Clinical Chemistry and Laboratory Medicine, Technische Universität Dresden, Dresden, Germany
[3]Department of Internal Medicine I, Gastroenterology, Hepatology, Endocrinology, and Metabolism, Innsbruck Medical University, Innsbruck, Austria.
[4]Department of Medicine (Rheumatology, Allergy & Immunology), Yale University School of Medicine, New Haven, CT, USA
[5]Department of Immunobiology, Yale University School of Medicine, New Haven, CT, USA
[6]Division of Rheumatology, Department of Medicine III, University Medical Centre and Faculty of Medicine Carl Gustav Carus at the TU Dresden, Germany
[7]Molecular Analysis - Mass Spectrometry, Center for Molecular and Cellular Bioengineering (CMCB), Technische Universität Dresden, Dresden, Germany

*Correspondence to:

Nikolai P. Jaschke MD, PhD
nikolai.jaschke@uniklinikum-dresden.de

This PDF file includes:

1. Tables S1 and S2
2. Supplementary figures S1-S7 with legends
3. References

1. Tables S1 and S2

RS_ID	experimental set-up	label	p-value	cytokine
rs11001560	IFNy_Borreliamix_PBMC_7days	1	6.97789531773793e-05	IFNy
rs11815201	IFNy_Borreliamix_PBMC_7days	1	0,002610734	IFNy
rs1569198	IFNy_Borreliamix_PBMC_7days	1	0,002824264	IFNy
rs1569198	IFNy_C.conidiaHK_WB_48h	2	0,038594554	IFNy
rs11815201	IFNy_C.conidiaHK_WB_48h	2	0,049473278	IFNy
rs1569198	IFNy_LPS_WB_48h	3	0,044593755	IFNy
rs11001560	IL1b_E.Coli_PBMC_24h	4	0,024668625	IL1b
rs11815201	IL1b_E.Coli_PBMC_24h	4	0,042949724	IL1b
rs11001560	IL6_C.albicanshyphae_PBMC_24h	5	0,02299243	IL6
rs11815201	IL6_C.albicanshyphae_PBMC_24h	5	0,03841946	IL6
rs1569198	IL6_C.albicanshyphae_PBMC_24h	5	0,049424669	IL6
rs11001548	IL6_Cryptococcus_PBMC_24h	6	0,033772885	IL6
rs11001553	IL6_Cryptococcus_PBMC_24h	6	0,033890907	IL6
rs11815201	IL6_LPS1ng_PBMC_24h	7	0,023874706	IL6
rs1569198	IL6_LPS1ng_PBMC_24h	7	0,032609547	IL6
rs11815201	TNFA_A.fumigatusconidiaSerum_PBMC_24h	8	0,019045995	TNFA
rs1569198	TNFA_A.fumigatusconidiaSerum_PBMC_24h	8	0,030568652	TNFA

Table S1. Genetic DKK1 variants associated with increased cytokine expression from the 500FG cohort study [1]. The row “label” refers to the numbers depicted in Figure 5A.

gene	sequence [forward= F and reverse=R]
GAPDH	F: AGCCACATCGCTCAGACAC<br>R: GCCCAATACGACCAAATCC
DKK1	F: AGCACCTTGGATGGGTATTC<br>R: CACACTTGACCTTCTTTCAGGAC
CXCL8	F: CTGGACCCCAAGGAAAACTG<br>R: TTCTCAGCCCTCTTCAAAAAC
IL1B	F: GTCCTGCGTGTTGAAAGATG<br>R: CTGCTTGAGAGGTGCTGATG
CXCL1	F: CACCCCAAGAACATCCAAAG<br>R: TAACTATGGGGGATGCAGGA
LEF1	F: CAAATAAAGTGCCCGTGGTG<br>R: CCTGGAGAAAAGTGCTCGTC
SOCS1	F: CTGGGATGCCGTGTTATTTTGT<br>R: TAAGCTGCTACAACAACCAGGG
SOCS3	F: TCAAGACCTTCAGCTCCAAGAG<br>R: AGATGTAATAGGCTCTTCTGGGG
LRP5	F: GACTCCTTCCCCGACTGTATC<br>R: CGCTGGCACACAAAATAGAC
LRP6	F: TCCTGATGGTATTGCTGTGG<br>R: CGGGGTTCCTCTAAGTCCTC
Actb	F: GATCTGGCACCACACCTTCT<br>R: GGGGTGTTGAAGGTCTCAAA
Dkk1	F: GCCTCCGATCATCAGACGGT<br>R: GCAGGTGTGGAGCCTAGAAG
Il1b	F: ACAAGGAGAACCAAGCAACG<br>R: GCCGTCTTTCATTACACAGG
Tnfa	F: CCTCTTCTCATTCCTGCTTGTG<br>R: CACTTGGTGGTTTGCTACGAC
Lef1	F: CAAATAAAGTGCCCGTGGTG<br>R: TCGTCGCTGTAGGTGATGAG
Opg	F: CCTTGCCCTGACCACTCTTA<br>R: ACACTGGGCTGCAATACACA

Table S2. Primer sequences used for qPCR analysis

2. Supplementary figures S1-S7 with legends

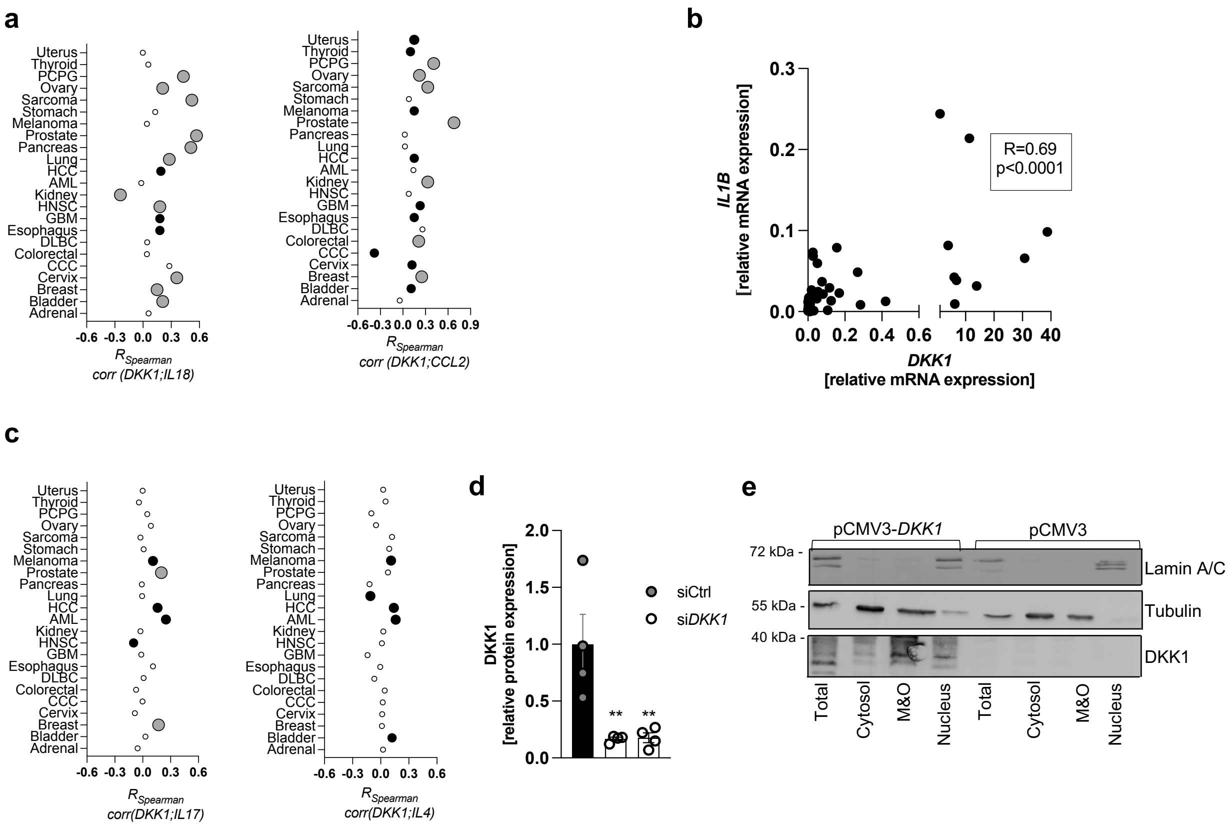

Fig.S1 (a,c) Correlations between transcript levels of *DKK1* and selected cytokines/chemokines (*IL18, CCL2, IL17, IL4*) in human tumor tissues visualized as bubble plots. Each dot is reflective of the Spearman correlation coefficient (R) noted for the association between DKK1 and cytokine/chemokine expression in the corresponding tissue (see X-axis). The size of the dot indicates the respective p-value, whereby larger dots reflect smaller (significant) p-values. Data was extracted from the Cancer Genome Atlas Project (TGCA). The following abbreviations are shown: PCPG= Pheochromocytoma and Paraganglioma, HCC= Hepatocellular Carcinoma, AML =Acute Myeloid Leukemia, HNSC= Head and Neck Squamous Cell Carcinoma, GBM= Glioblastoma Multiforme, DLBC = Diffuse Large B-Cell Lymphoma, CCC= Cholangiocellular Carcinoma (b) Correlation between *DKK1* and *IL1B* mRNA expression in primary prostate cancer tissue. Results were generated using a commercially available prostate cancer cDNA array and visualized by Prism. Spearman's correlation coefficient (R) is shown (d) DKK1 protein levels in DKK1-competent and -deficient PC3 cells determined by immunoblotting. Band intensities were quantified by ImageJ (n=4/genotype; both DKK1-targeting siRNAs are shown) (e) Representative immunoblot from

T47D subcellular fractions following transfection of a plasmid encoding the full open-reading frame (ORF) of human DKK1 (pCMV3-DKK1) or an empty backbone vector (pCMV3). ****$p<0.0001$. Data is shown as mean ± SEM [(d) one-way ANOVA with Holm-Sidak's post-hoc test]

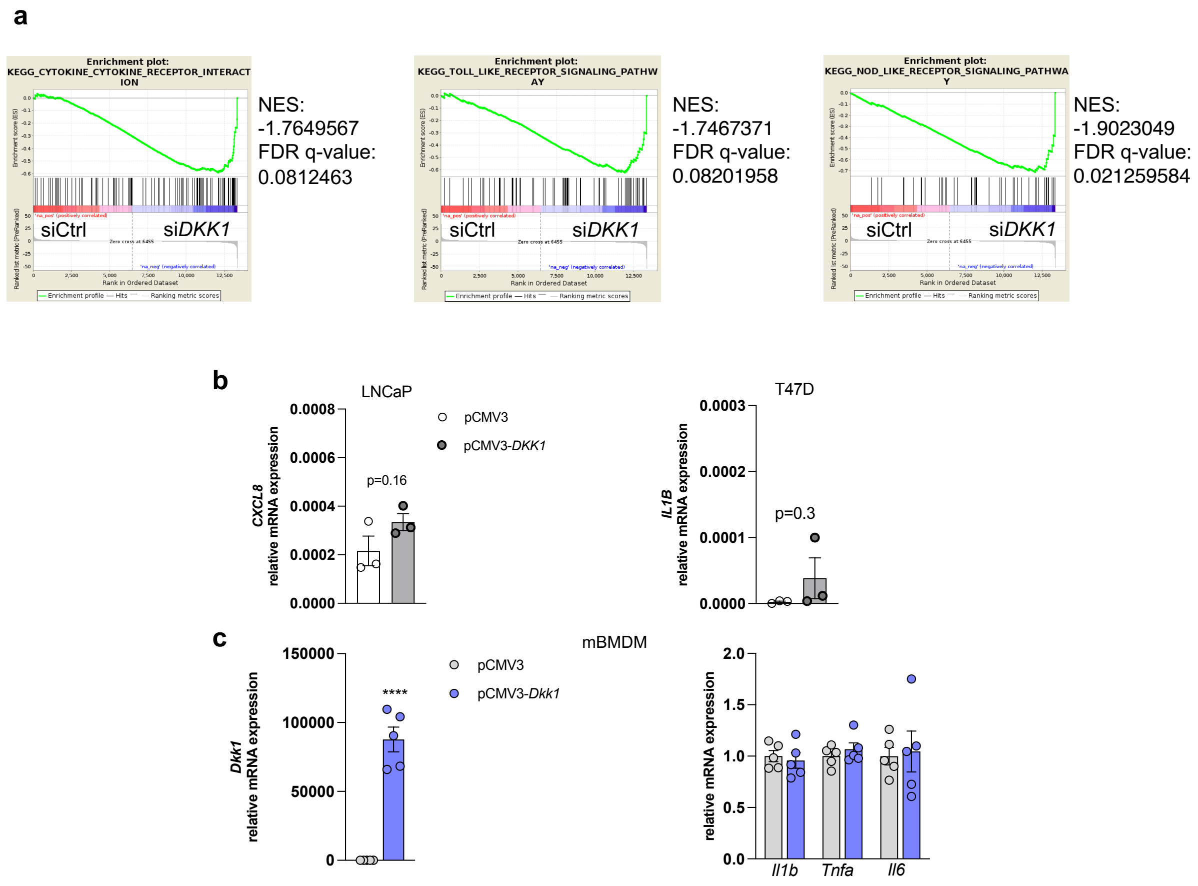

Fig.S2 (a) Exemplary enrichment plots from RNA sequencing results of DKK1-competent (siCtrl) and -deficient (siDKK1) PC3 cells (b) *CXCL8* and *IL1B* mRNA levels in human LNCaP and T47D tumor cells following transfection of a plasmid encoding the full ORF of human DKK1 (pCMV3-DKK1) or an empty backbone vector (pCMV3) (n=3/genotype) (c) *DKK1* (left panel) and inflammatory cytokine transcript levels (right panel) in murine bone marrow-derived macrophages (mBMDM) transfected with a plasmid encoding the full ORF of murine DKK1 (pCMV3-Dkk1) or an empty backbone vector (pCMV3) (n=4/genotype). ****$p<0.0001$. Data is shown as mean ± SEM. [(b,c) two-tailed, unpaired student's t-test]

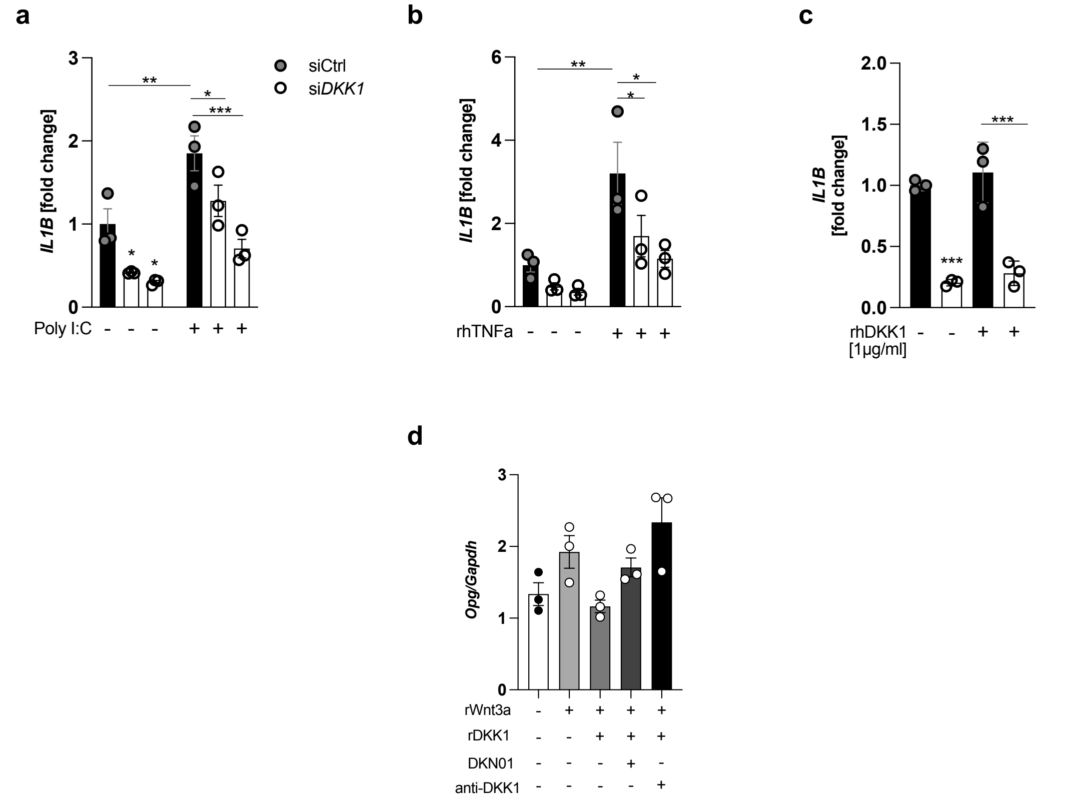

Fig.S3 (a,b) *IL1B* transcript levels in DKK1-competent and -deficient PC3 cells exposed to high molecular weight Poly I:C (TLR3 agonist) or recombinant human tumor necrosis factor alpha (TNFa) for 24h (n=3/genotype and condition) (c) *IL1B* transcript levels in DKK1-deficient cells following 24h exposure to recombinant human DKK1 from R&D (1µg/ml) (n=3) (d) mRNA levels of the Wnt-target gene osteoprotegerin (*Opg*) in primary murine osteoblasts following treatment with recombinant Wnt3a (200ng/ml) with or without recombinant DKK1 (250ng/ml) and DKK1-neutralizing antibodies (DKN01 and anti-DKK1, respectively, both at 10µg/ml) (n=3/condition).*p<0.05; **p<0.01, ***p<0.001, ****p<0.0001. Data is expressed as mean ± SEM. [(A-D) one-way ANOVA with Holm-Sidak's post-hoc test]

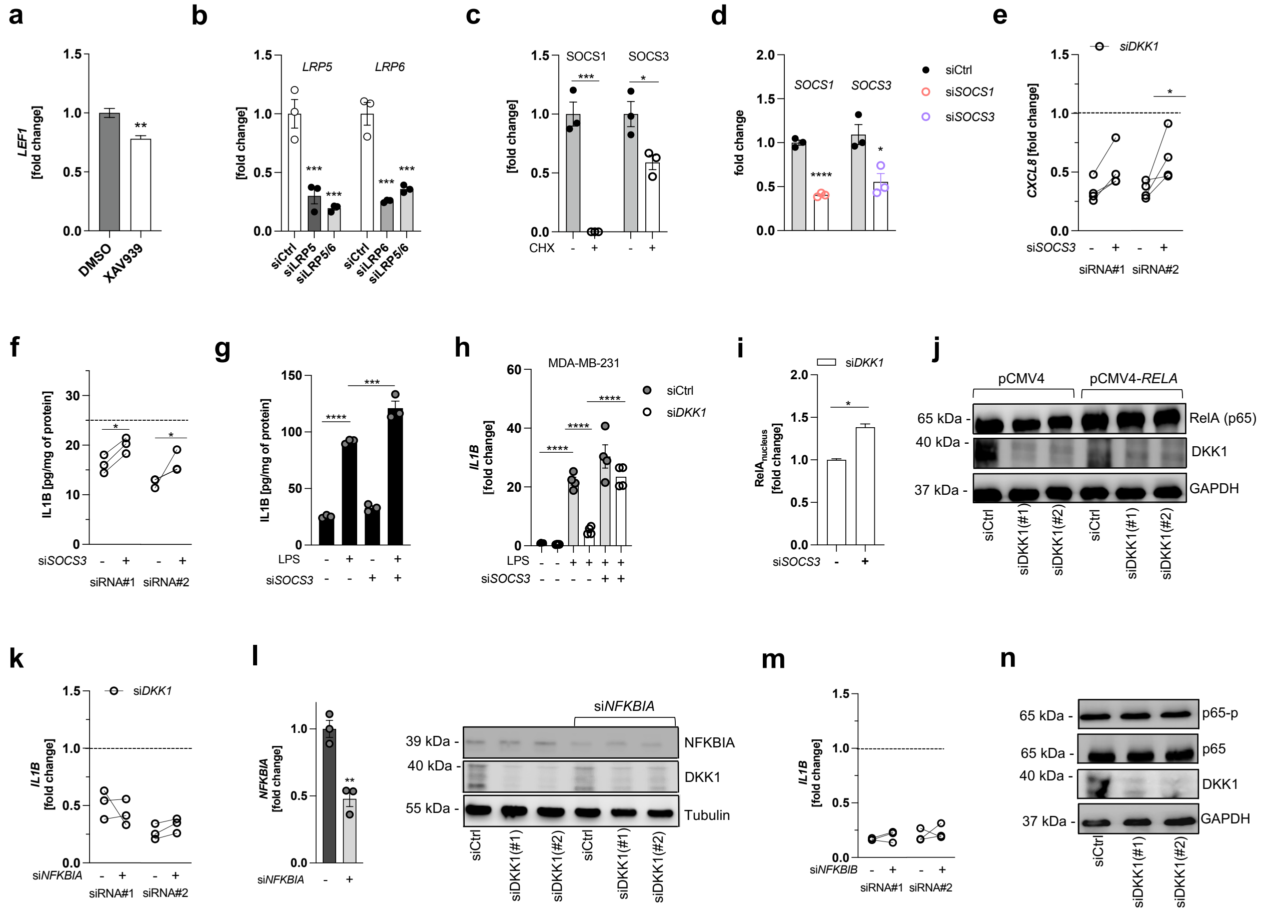

Fig.S4 (a) Transcript levels of the Wnt-target gene *LEF1* in PC3 cells following 24h exposure to XAV939 (5µM) (n=3/condition) (b) *LRP5* and *LRP6* mRNA expression in PC3 cells 48h after exposure to LRP5/6-targeting siRNAs (n=3) (c) Quantification of SOCS1 and SOCS3 protein levels from Fig. 4g (n=3) (d) *SOCS1* and *SOCS3* mRNA expression 48h following exposure to SOCS1/3-specific siRNAs (n=3) (e) *CXCL8* transcript levels in DKK1-deficient cells following SOCS3 knockdown. Results for two independent DKK1-targeting siRNAs are shown (siRNA#1 and siRNA#2, respectively). The dashed line corresponds to *CXCL8* expression of DMSO-treated PC3 cells transfected with non-targeting control oligonucleotides (siCtrl) (n=3) (f) IL1B protein abundance in total cell lysates from DKK1-deficient PC3 cells with (+) or without (-) SOCS3 knockdown (n=3) (g) IL1B protein levels in total cell lysates of wildtype (DKK1-competent) PC3 cells with (+) or without (-) SOCS3 knockdown exposed to LPS (1µg/ml) or water for 24h (n=3) (h) Transcript levels of *IL1B* in DKK1-competent and -deficient MDA-MB-231 cells with (+) or without (-) SOCS3 knockdown treated with LPS (1µg/ml) or water for 24h (n=4/condition) (i) Quantification of nuclear RelA abundance in DKK1-deficient PC3 cells with (+) or without (-) SOCS3 knockdown. Results from two biologically independent replicates are shown (refers to Figure 4m) (j) Representative RelA immunoblot in DKK1-competent or -deficient PC3 cells transfected with a plasmid encoding the full open-reading frame of human Rela (pCMV4-RelA) or an empty backbone vector (pCMV4) (refers to Fig. 4n) (k) *IL1B* mRNA

expression in DKK1-deficient cells with (+) or without (-) IkBa knockdown (si*NFKBIA*). Results for two independent DKK1-targeting siRNAs are shown (siRNA#1 and siRNA#2). The dashed line corresponds to *IL1B* expression of DMSO-treated PC3 cells transfected with non-targeting control oligonucleotides (siCtrl) (n=3/genotype) (l) Confirmation of IkBa knockdown at mRNA and protein level (m) *IL1B* mRNA expression in DKK1-deficient cells with (+) or without (-) IkBb knockdown (siNFKBIB) (n=3/genotype) (n) RelA phosphorylation in DKK1-competent and -deficient PC3 cells *p<0.05; **p<0.01, ***p<0.001, ****p<0.0001. Data is expressed as mean ± SEM [(a,c-g and i,l): unpaired, two-tailed student's t-test, (b,g,h) one-way ANOVA with Holm-Sidak's post-hoc test].

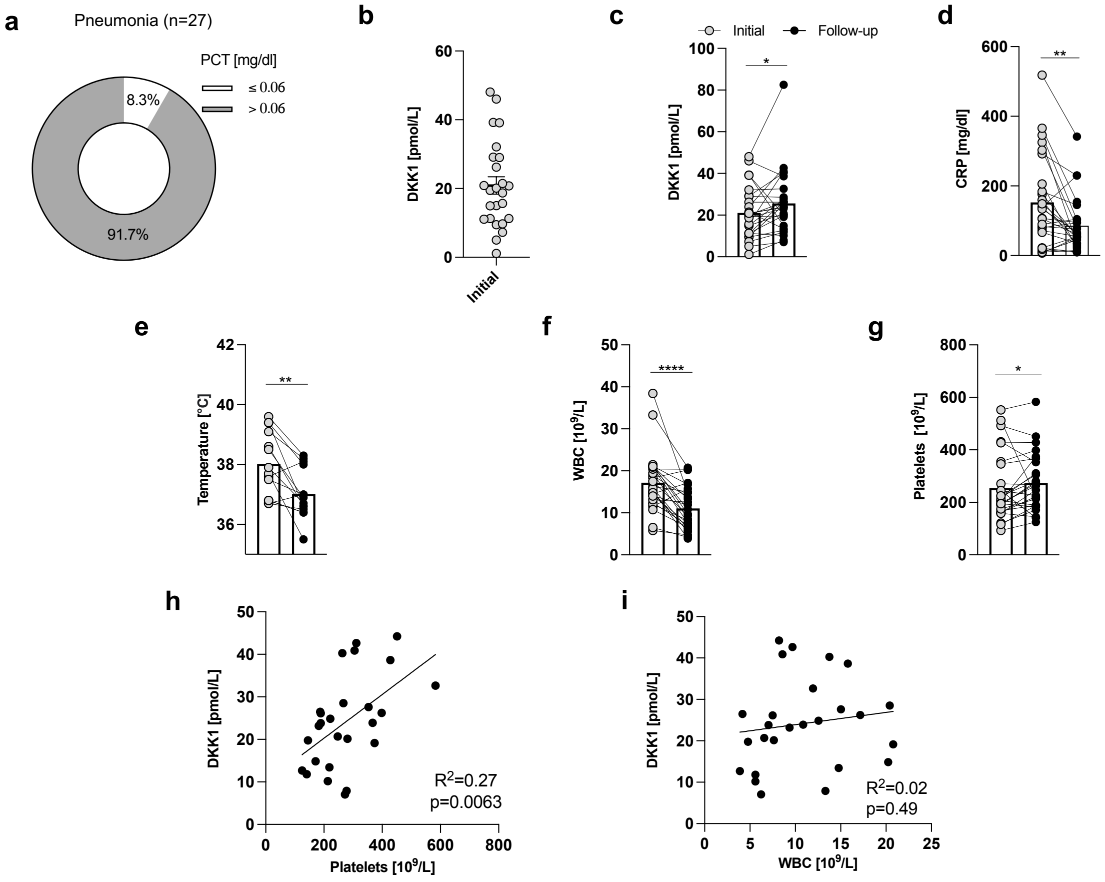

Fig.S5 (a) Relative percentage of hospitalized patients with acute pneumonia (n=27) displaying either elevated (>0.06 mg/ml) or normal (<0.06 mg/dl) procalcitonin (PCT) serum levels (b) Circulating DKK1 serum levels in the same patients during the acute phase of pneumonia (= day 1 or 2 of hospitalization; referred to as "initial") determined by ELISA. Note the high interindividual variability (c) Change in circulating DKK1 levels across the disease trajectory. The first ("initial") and last available measure of each patient are shown, the latter referred to as "follow-up" (>day 3 and <day 7 of hospitalization) (d-g) Corresponding changes in c-reactive protein (CRP) serum levels, white blood cell count (WBC), body temperature and platelet numbers (h,i) Association between platelet or white blood cell counts (WBC) and DKK1

serum levels at follow-up determined by linear regression analysis. Data is expressed as mean ± SEM. [c-g) Wilcoxon-signed-rank test (h,i) Least square fit (linear regression)]

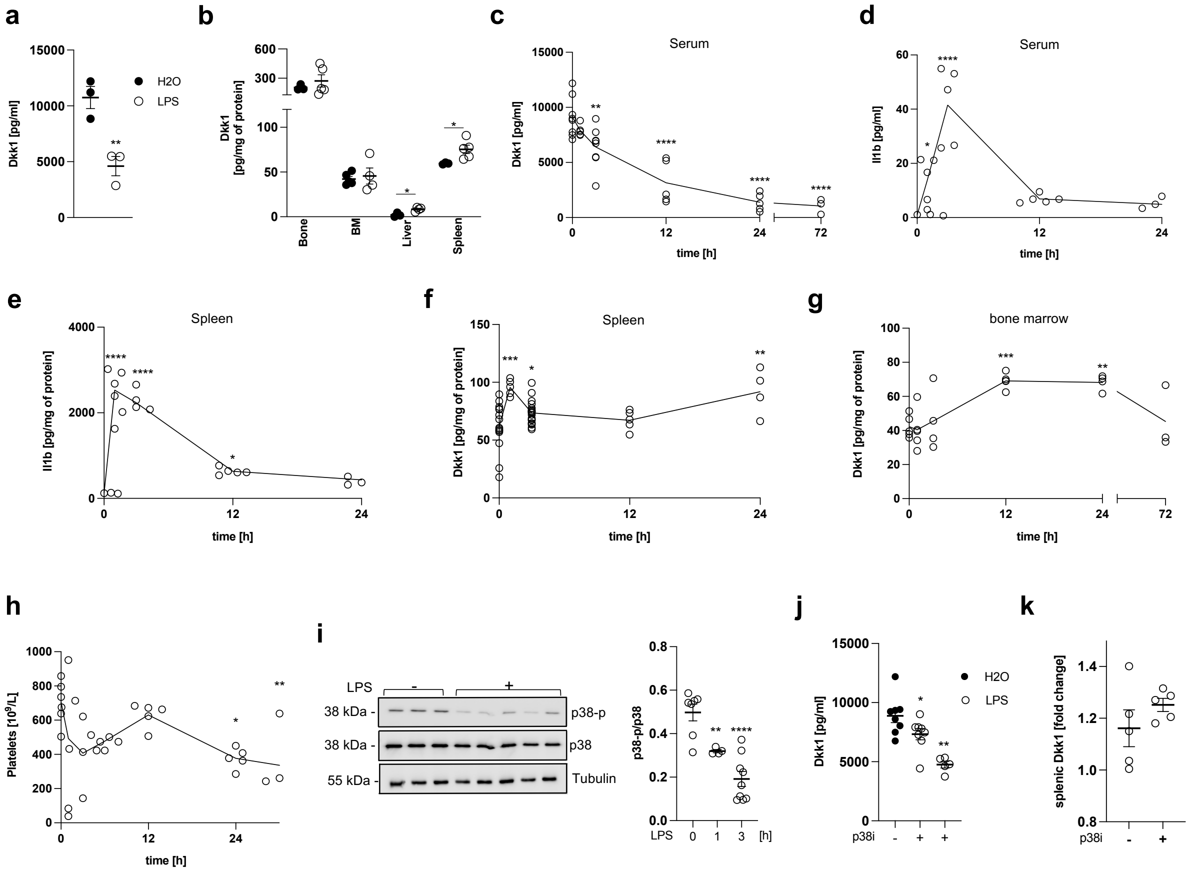

Fig S6 (a) Dkk1 serum levels 3h following LPS injection (5 mg/kg) in C57BL/6J wildtype mice (n=3) (b) Corresponding Dkk1 tissue levels in different organs determined by ELISA (n=4-5) (c) Circulating Dkk1 levels in C57BL/6J mice at various time points following LPS administration (n=4-8/time point) (d,e) Il1b serum and splenic tissue levels in the same mice measured by ELISA (n=5/time point) (f,g) Corresponding Dkk1 protein levels in the spleen and bone marrow (n=4-15/time point) (h) Platelet kinetics (n=5/time point) (i) Representative p38 immunoblot from spleen lysates of C57BL/6J wildtype mice at baseline and 3h following LPS treatment (left panel). Band intensities were quantified by ImageJ (right panel) (j) Circulating Dkk1 concentration in C57BL/6J wildtype mice treated with or without the pharmacological p38 inhibitor ralimetinib dimesylate (LY22288220) at 5 mg/kg prior to LPS or water exposure for 1h (n=5-8/group) (k) Fold change of Dkk1 protein abundance (normalized to vehicle treated controls) in spleen lysates from the same mice (n=5/group)**.** Data is expressed as mean ± SEM. [a,b) two-tailed, unpaired, student's t-test, (c-j) one-way ANOVA with Holm-Sidak's post-hoc test.]

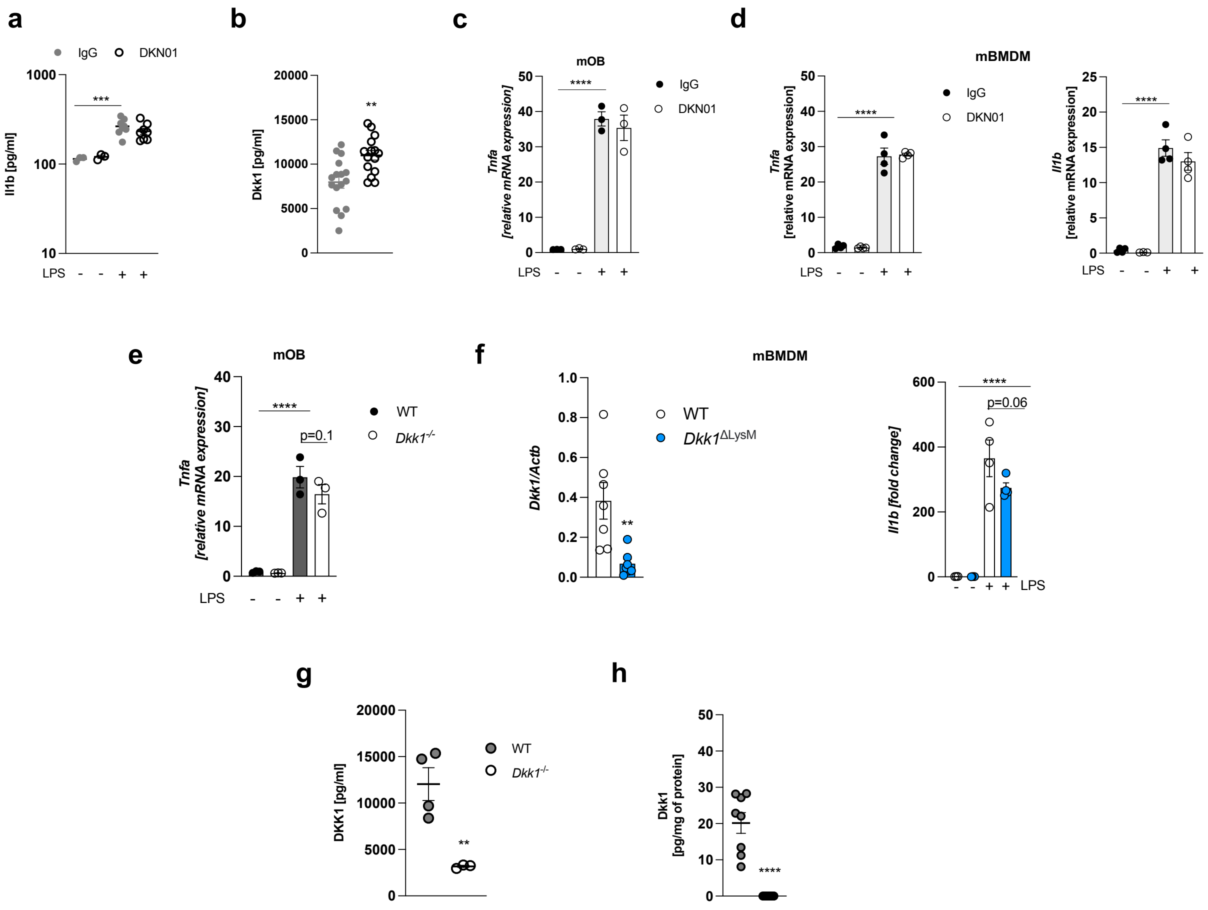

Fig.S7 (a) Circulating Il1b levels in C57BL/6J mice treated with LPS (5mg/kg). Eighteen hours prior to being challenged with LPS, mice received a single injection of a Dkk1-neutralizing antibody (DKN01, 10 mg/kg) or an equivalent dose of isotype control antibody (murine IgG) (n=3-7/group) (b) Circulating Dkk1 levels in mice treated with DKN01 or isotype control (IgG). Successful Dkk1 neutralization is indicated by elevated Dkk1 levels as previously reported[3] (n=15-16/group) (c) Tnf alpha (*Tnfa*) mRNA expression in wildtype osteoblasts treated with LPS (1µg/m) for 6h. Cells were exposed to a Dkk1-neutralizing antibody (DKN01, 10µg/ml) or isotype control (murine IgG) 18h prior to being challenged with LPS (n=4/group) (d) *Tnfa* and *Il1b* transcript levels in murine bone marrow derived macrophages (mBMDM) treated with LPS (0.1µg/ml) for 6h. Cells were exposed to DKN01 (10µg/ml) or isotype control (murine IgG) 18h prior to being challenged with LPS (n=4/group) (e) *Tnfa* mRNA expression in Dkk1 knock-out osteoblasts ($Dkk1^{-/-}$) or wildtype littermate controls ($Dkk1^{fl/fl}$) following 6h exposure to LPS (1µg/ml) (n=3/group) (f) *Dkk1* transcript levels in mBMDM isolated from mice with myeloid cell-specific Dkk1 deletion ($Dkk1^{fl/fl}$;LysM:cre, referred to as $Dkk1^{\Delta LysM}$) or wildtype littermate controls. The right panel shows *Il1b* mRNA expression in mBMDM from the same mice following 6h exposure to LPS (50 ng/ml) (n=3/group) (g) Circulating Dkk1 levels in mice with ($Dkk1^{-/-}$) or without ($Dkk1^{fl/fl}$; referred to as wildtype, "WT") global Dkk1 deletion determined by ELISA (n=3-4/group) (h) Dkk1 protein levels bone lysates from the same mice (n=8/group).

*p<0.05; **p<0.01, ***p<0.001, ****p<0.0001. Data is expressed as mean ± SEM. [(a, c-f): one-way ANOVA with Holm-Sidak's post-hoc test, (b, g, h) unpaired, two-tailed student's t-test].

3. References

1. Li, Y.*, et al.* A Functional Genomics Approach to Understand Variation in Cytokine Production in Humans. *Cell* **167**, 1099-1110.e1014 (2016).
2. Pietzner, M.*, et al.* Mapping the proteo-genomic convergence of human diseases. *Science* **374**, eabj1541 (2021).
3. Haas, M.S.*, et al.* mDKN-01, a Novel Anti-DKK1 mAb, Enhances Innate Immune Responses in the Tumor Microenvironment. *Mol Cancer Res* **19**, 717-725 (2021).
