# Supplementary material for: Dickkopf1 fuels inflammatory cytokine responses
Source: Commun Biol. 2022 Dec 20;5:1391. doi: 10.1038/s42003-022-04368-8 (PMC9765382; doi:10.1038/s42003-022-04368-8)
Supplement: Supplementary file 2 — Description of additional Supplementary Files [file 42003_2022_4368_MOESM2_ESM.docx]

**Description of Additional Supplementary Files**

File name: Supplementary Data 1

Description: Links between DKK1-associated protein quantitative trait loci (pQTL) and cytokine expression in humans. Data were extracted from a publicly available platform (https://www.omicscience.org/apps/pgwas/)

File name: Supplementary Data 2

Description: Source data behind all graphs from main and supplementary figures of the paper.

File name: Supplementary Data 3

Description: Western Blot source data (uncropped blots).
