# Supplementary material for: Dickkopf1 fuels inflammatory cytokine responses
Source: Commun Biol. 2022 Dec 20;5:1391. doi: 10.1038/s42003-022-04368-8 (PMC9765382; doi:10.1038/s42003-022-04368-8)
Supplement: Supplementary file 6 — Reporting Summary [file 42003_2022_4368_MOESM6_ESM.pdf]

nature portfolio

Corresponding author(s): Nikolai P. Jaschke MD, PhD

Last updated by author(s): Dec 2, 2022

Reporting Summary

Nature Portfolio wishes to improve the reproducibility of the work that we publish. This form provides structure for consistency and transparency in reporting. For further information on Nature Portfolio policies, see our Editorial Policies and the Editorial Policy Checklist.

Statistics

For all statistical analyses, confirm that the following items are present in the figure legend, table legend, main text, or Methods section.

n/a	Confirmed	
☐	☒	The exact sample size (*n*) for each experimental group/condition, given as a discrete number and unit of measurement
☐	☒	A statement on whether measurements were taken from distinct samples or whether the same sample was measured repeatedly
☐	☒	The statistical test(s) used AND whether they are one- or two-sided<br>*Only common tests should be described solely by name; describe more complex techniques in the Methods section.*
☒	☐	A description of all covariates tested
☐	☒	A description of any assumptions or corrections, such as tests of normality and adjustment for multiple comparisons
☐	☒	A full description of the statistical parameters including central tendency (e.g. means) or other basic estimates (e.g. regression coefficient) AND variation (e.g. standard deviation) or associated estimates of uncertainty (e.g. confidence intervals)
☐	☒	For null hypothesis testing, the test statistic (e.g. *F*, *t*, *r*) with confidence intervals, effect sizes, degrees of freedom and *P* value noted<br>*Give P values as exact values whenever suitable.*
☒	☐	For Bayesian analysis, information on the choice of priors and Markov chain Monte Carlo settings
☒	☐	For hierarchical and complex designs, identification of the appropriate level for tests and full reporting of outcomes
☐	☒	Estimates of effect sizes (e.g. Cohen's *d*, Pearson's *r*), indicating how they were calculated

Our web collection on statistics for biologists contains articles on many of the points above.

Software and code

Policy information about availability of computer code

Data collection	StepOne Software, Omega (BMG Labtech), Microsoft Excel, ImageJ
Data analysis	Statistical Analysis were performed using Graphpad V9. Large genetic datasets and bionformatic analysis were performed using R and the respective packages listed in the "Methods" section.

For manuscripts utilizing custom algorithms or software that are central to the research but not yet described in published literature, software must be made available to editors and reviewers. We strongly encourage code deposition in a community repository (e.g. GitHub). See the Nature Portfolio guidelines for submitting code & software for further information.

Data

Policy information about availability of data

All manuscripts must include a data availability statement. This statement should provide the following information, where applicable:

- Accession codes, unique identifiers, or web links for publicly available datasets
- A description of any restrictions on data availability
- For clinical datasets or third party data, please ensure that the statement adheres to our policy

RNAseq data from human tumor tissues were extracted from the GEPIA webserver (http://gepia.cancer-pku.cn). RNA sequencing data from human tumor cells is available at Gene Expression Omnibus (GEO) via accession number GSE217231 (https://www.ncbi.nlm.nih.gov/geo/query/acc.cgi?acc=GSE217231). All other data datasets involving humans were downloaded from publicly available platforms as cited in the manuscript. Source data is provided in the respective supplementary data files.

Field-specific reporting

Please select the one below that is the best fit for your research. If you are not sure, read the appropriate sections before making your selection.

☒ Life sciences ☐ Behavioural & social sciences ☐ Ecological, evolutionary & environmental sciences

For a reference copy of the document with all sections, see nature.com/documents/nr-reporting-summary-flat.pdf

Life sciences study design

All studies must disclose on these points even when the disclosure is negative.

Sample size	Sample sizes were chosen based on experience from previous experiments of the authors or the published literature.
Data exclusions	Grubbs Test (alpha=0.05) allowed for the exclusion of one statistically significant outlier per dataset. During LPS mortality studies, one mouse was excluded from analysis due to an insufficient biological response (reflected by a lack of change in body temperature).
Replication	Key results were confirmed by two independent scientists. Replication of these findings were all successful. Reagents from independent suppliers were used to validate unexpected observations (e.g. rDKK1)
Randomization	Animals were randomly assigned to experimental groups.
Blinding	For most animal experiments, scientists were not blinded to genotypes and treatments due to practical reasons. Data collection and analyses were performed in a blinded fashion.

Reporting for specific materials, systems and methods

We require information from authors about some types of materials, experimental systems and methods used in many studies. Here, indicate whether each material, system or method listed is relevant to your study. If you are not sure if a list item applies to your research, read the appropriate section before selecting a response.

Materials & experimental systems

n/a	Involved in the study	
☐	☒	Antibodies
☐	☒	Eukaryotic cell lines
☒	☐	Palaeontology and archaeology
☐	☒	Animals and other organisms
☐	☒	Human research participants
☒	☐	Clinical data
☒	☐	Dual use research of concern

Methods

n/a	Involved in the study	
☒	☐	ChIP-seq
☒	☐	Flow cytometry
☒	☐	MRI-based neuroimaging

Antibodies

Antibodies used	anti-beta-Tubulin (1:1000, Cell Signaling Technology, #2146), anti-DKK1 (1:500, R&D, #AF1096), anti-Lamin A/C (1:1000, Santa Cruz Biotechnology, #sc-376248), anti-SOCS1 (1:500, Cell Signaling Technology, #3950), anti-SOCS3 (1:500, Santa Cruz Biotechnology, #sc-73045), anti-SOCS3 (1:500, Cell Signaling Technology, #52113), anti-RelA/p65 (1:1000, Cell Signaling Technology, #8242), anti-RelA/p65-phospho (1:1000, Cell Signaling Technology, #3033), anti-p38 (1:1000, Cell Signaling Technology, #9212), anti-p38-phospho (1:1000, Cell Signaling Technology, #4511)
Validation	All antibodies tested produced a single band at the expected molecular size and were validated by the respective suppliers using knock-out approaches (see R&D, CST and Santa Cruz Websites). The specificity of the anit-DKK1 antibody was validated in-house using siRNA to ensure that the various bands detected are all reflective of DKK1 protein.

Eukaryotic cell lines

Policy information about cell lines

Cell line source(s)	Human cancer cell lines (PC3, MDAMB231, MCF7, T47D and SaoS2) were obtained from the German Collection of Microorganisms and Cell Cultures (DSMZ). Gastrointestinal cancer cell lines (LoVo, Caco-2, Huh-7, HepG2) were kindly provided by Prof. Zeissig (TU Dresden), while DU145 and LNCaP cells were a gift from Prof. Dubrovska (TU Dresden).
Authentication	The genetic authenticity of each cell line was verified at the DSMZ (German Collection of Microorganisms and Cell Cultures) where short tandem repeat profiling was matched with known profiles.

Mycoplasma contamination | Mycoplasma contamination was not regularly assayed

Commonly misidentified lines (See ICLAC register) | n/a

Animals and other organisms

Policy information about studies involving animals; ARRIVE guidelines recommended for reporting animal research

Laboratory animals: C57BL/6J Dkk1fl/fl mice with loxP sites flanking exons 1 and 2 of the Dkk1 locus were crossed with C57BL/6J Rosa26-CreERT2 mice to obtain Dkk1fl/fl:Rosa26-CreERT2 allowing for tamoxifen-inducible global deletion of Dkk1 (Dkk1-/-). Cre-negative littermates were used as controls (referred to as wildtype; WT). Mice with osteoblast/osteocyte-specific Dkk1 knock-out (Dkk1ΔDmp1) were generated by crossing Dkk1fl/fl mice with Dmp1:Cre transgenic animals , while Dkk1fl/fl mice were crossed with LysM:Cre transgenic animals to yield animals with myeloid cell-specific Dkk1 deletion. Cre-negative littermates served as controls. C57BL/6J wildtype mice were purchased from Janvier at the age of 6-8 weeks and used following an acclimatization period of 2 weeks. All mice were used for experimental procedures at the age of 8-10 weeks. For experiments involving circulating cytokine read-outs, only male mice were used to correct for sex differences in the inflammartory response. LPS-induced mortality in WT vs. DKK1 -/- mice was evaluted in both sexes and data was pooled.

Wild animals: n/a

Field-collected samples: All mice were housed in groups of 3-6 at the animal facility of the Technical University of Dresden and kept on a 12h light:dark cycle. Animals had ad libitum access to food and water throughout all studies and received bedding material.

Ethics oversight: All animal experiments were performed according to local institutional guidelines and approved by the Landesdirektion Sachsen.

Note that full information on the approval of the study protocol must also be provided in the manuscript.

Human research participants

Policy information about studies involving human research participants

Population characteristics: Patient characteristics from the pneumonia cohort are described in the manuscript.

Recruitment: Patients (age >18 und <90 years) admitted to the University Hospital Dresden with a clinical and/or radiographical diagnosis of pneumonia were recruited for the study (n=27). No other prespecified inclusion criteria were applied. Individuals who had received anitbiotics prior to hospital admission were excluded from the study. Repeated blood sampling was performed by trained hospital stuff.

Ethics oversight: This study was approved by the local institutional review board (EK 191052016)

Note that full information on the approval of the study protocol must also be provided in the manuscript.
